# Lung Carcinoma Presenting as a Superior Vena Cava Syndrome, Burnt and Twice Reborn as Adrenal and Facial Tumors

**DOI:** 10.7759/cureus.5746

**Published:** 2019-09-24

**Authors:** Kate Young, Eitan Friedman

**Affiliations:** 1 Internal Medicine, Ross University School of Medicine, Tampa, USA; 2 Oncology/hematology, United Oncology Medical Associates, Miami, USA

**Keywords:** neuroendocrine tumors, intracranial metastasis, chemo, immuno-oncology, non-small cell lung cancer, genetic profile, adrenal metastasis, oligo-metastatic, paraneoplastic syndrome, molecular

## Abstract

Eighty-five percent of all lung cancers are Non-Small-Cell Lung Carcinoma (NSCLC) with common sites of metastasis to the adrenal glands and liver. Onset is insidious, and seventy-five percent of patients have either regional or distant metastases at initial presentation. The five-year relative survival rate is four and a half percent with a distantly spread disease based on recent studies. Here we present a unique case of a ten-year survival with NSCLC initially presenting as a Superior Vena Cava Syndrome and reoccurring with adrenal gland, bone, and CNS lesions. The patient presented with SVC caused by lung cancer and underwent chemo and radiotherapy with complete response in 2010. Five years later, the same cancer returned disguised as an adrenal tumor. In 2017, the patient came in with facial neuropathy, shooting pains, sinus headaches, eyelid concerns, and active tumoral activity was detected in the middle cranial fossa, involving parotid glands and the vertebral column. Craniotomy revealed a metastatic poorly differentiated adenocarcinoma that extended through foramen ovale and rotundum to the infratemporal fossa and caused left-sided facial paralysis, hearing loss and numbness in CN V2 - V3 distribution. Considering that the patient has experienced several recurrences of disease on standard protocols and is not a candidate for targeted molecular therapies, an immunotherapy trial was suggested as the next step. The natural history of this disease is remarkable in terms of metastatic sites, paraneoplastic manifestations, and a substantially prolonged lifespan. Thus, more studies of similar cases will advance our understanding of tumor genetics and immunotherapy allowing the greater benefit to future patients.

## Introduction

The onset of lung cancer is insidious, and 75% of patients have either regional or distant metastases at initial presentation. The five-year relative survival rate is 4.5% with a distantly spread disease based on recent studies [1]. 

Most paraneoplastic syndromes and SVC obstruction are caused by Small Cell Carcinoma (SCLC), with SCLC and Non-Small Cell Lung Carcinoma (NSCLC) cases being 10% and 1.7% respectively. SCLC occurs in smokers, metastasizes early, and bears the worst prognosis. SCLC tends to be centrally located and grows at double the rate of NSCLC. NSCLC, especially adenocarcinoma, occurs most often in female non-smokers and tends to be located peripherally. As NSCLC is the more common lung cancer in the overall population, its higher incidence makes NSCLC accountable for 50% of SVC syndrome occurrences while Small Cell Lung Carcinoma, Lymphoma, and Metastatic lesions account for 25%, 10%, and 10% respectively [2, 3].

Here we present a unique case of ten-year survival with NSCLC initially presenting as a Superior Vena Cava Syndrome (SVCS) and reoccurring with adrenal and intracranial nerve lesions, a pattern that is more representative of Small Cell Lung Carcinoma than Non-Small Cell Lung Carcinoma.

## Case presentation

**Figure 1:**
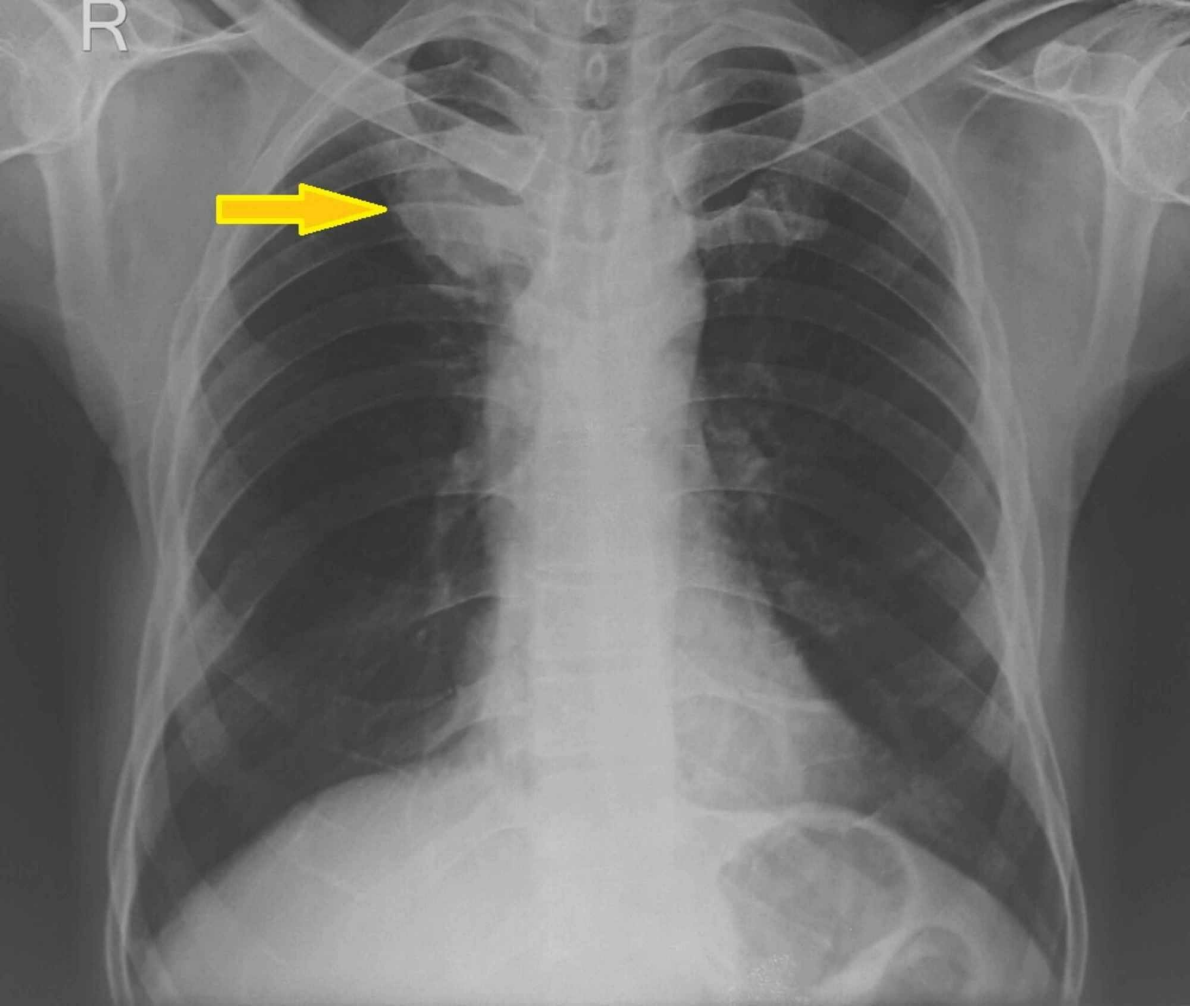
Chest X-ray showing NSCLC lesion causing SVC syndrome. Note a poorly defined soft tissue mass in the right upper zone [9].

**Figure 2:**
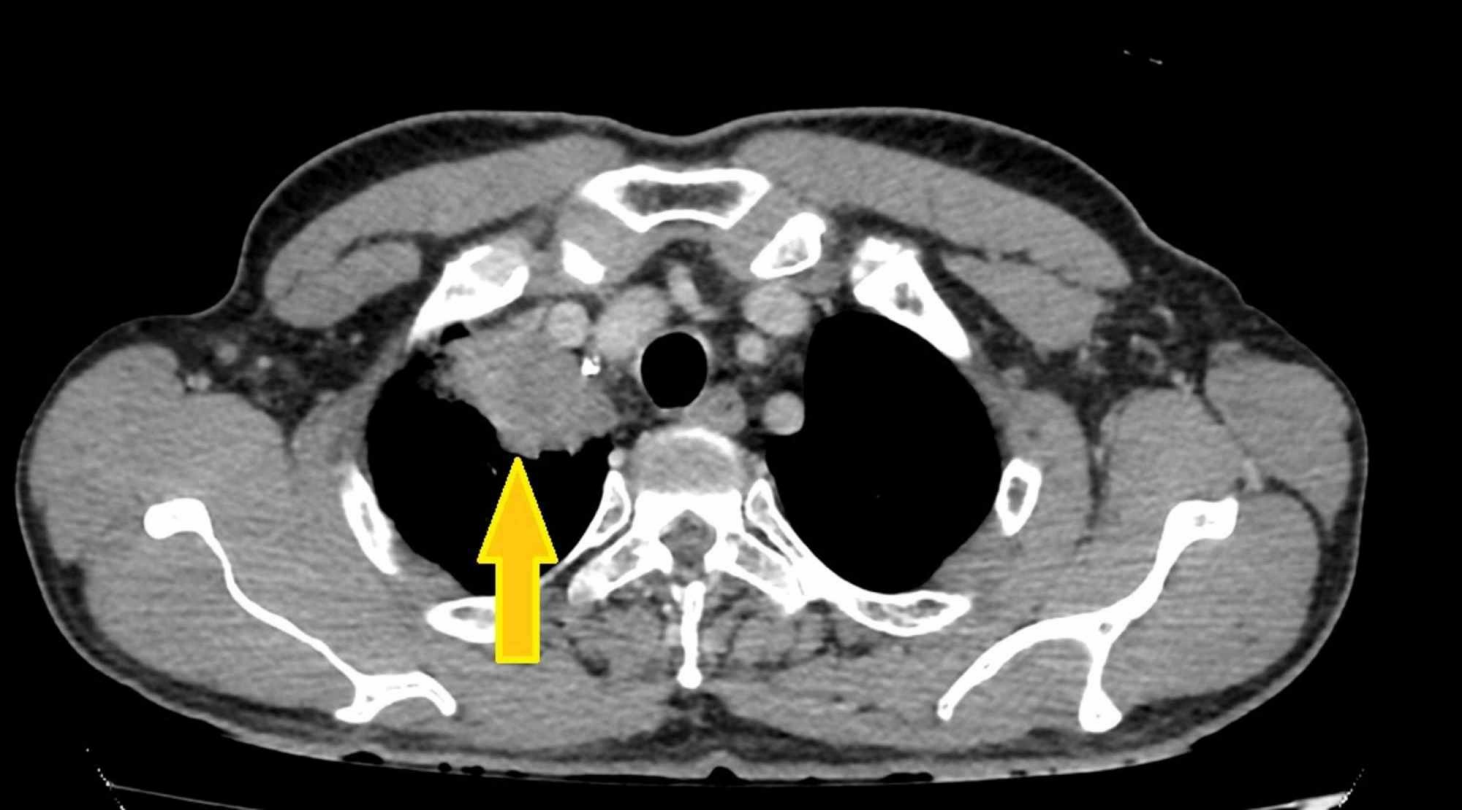
CT showing the lesion compressing adjacent structures. CT is the modality of choice for this malignant presentation [9].

**Figure 3:**
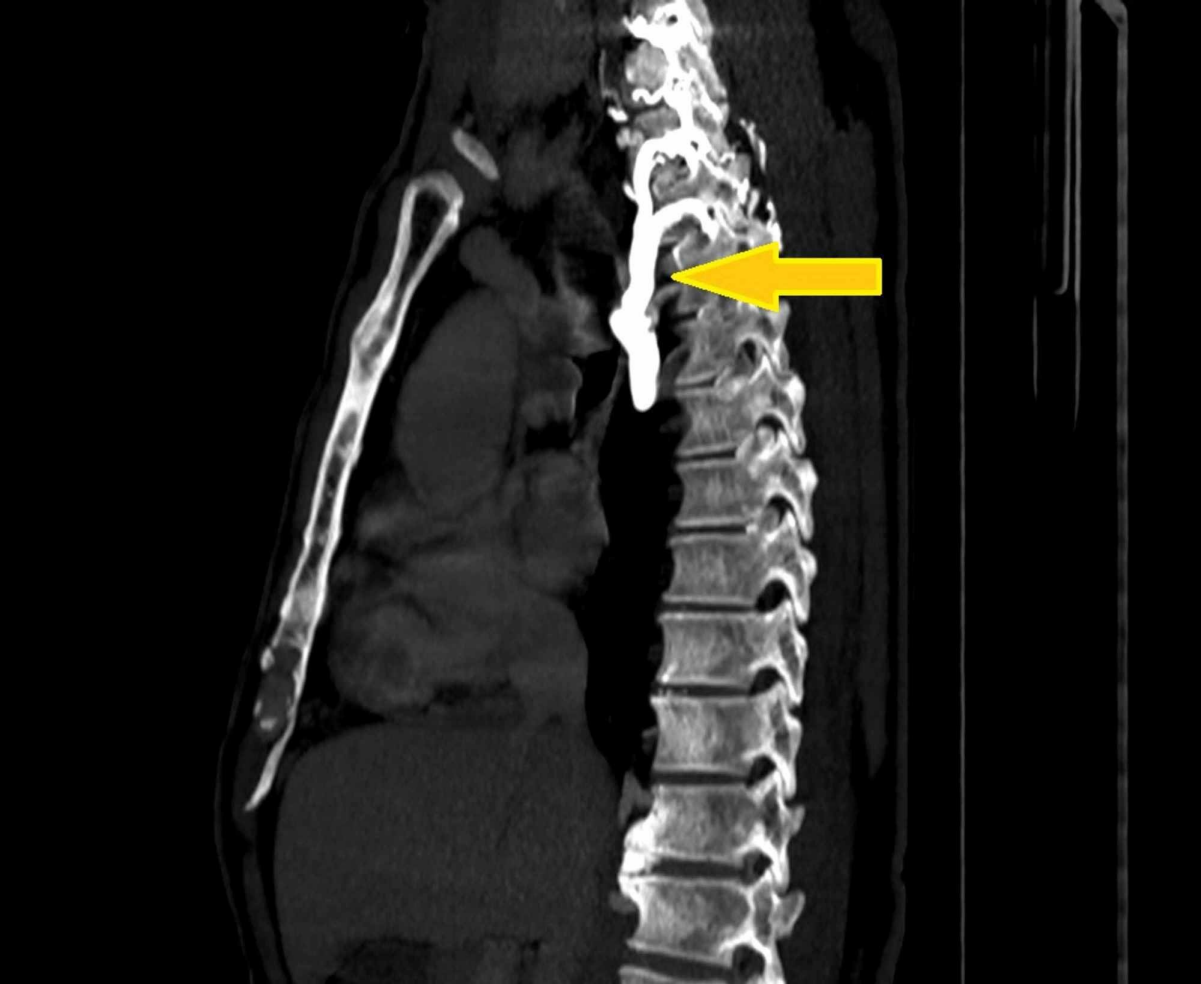
Collateral circulation in SVC syndrome. Note the prominent right superior intercostal vein, its tributaries and azygos vein with vivid enhancement, consistent with collateral formation due to obstruction of pre-azygos segment of SVC. The prominent raised torturous veins on the chest and abdomen often do not resolve after curative cancer treatment [9].

The patient is a 60-year-old Caucasian male with no significant past medical, social, or family history who presented with dyspnea, chest pain, neck swelling, and venous congestion consistent with Superior Vena Cava Syndrome [4, 5]. A lung mass was discovered in 2010 (at the age of 50) and a diagnosis of adenocarcinoma, a type of a non-small cell lung carcinoma, was made. The mass was negative for ALK/KRAS/EGFR/BRAF mutations [6, 7, 8] that are typically positive in NSCLC and negative in SCLC. See Figure 1, Figure 2, and Figure 3 for diagnostic imaging [9] related to physical exam findings in a similar case (courtesy of Dr.Prashant Mudgal, Radiopedia.org, rID:36497).

He underwent chemotherapy with Cisplatin/Paclitaxel and radiotherapy with complete response. In 2015, a symptomatic isolated left adrenal mass was discovered, resected, and treated with radiotherapy with complete resolution of symptoms. The excised adrenal mass was positive for TTF confirming NSCLC to be source of metastasis [10].

**Figure 4:**
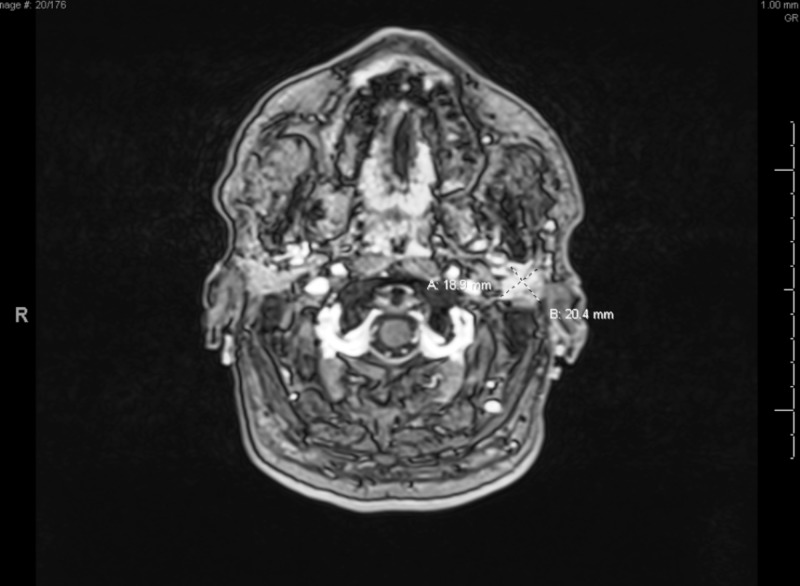
CT image of the patient's symptomatic adenocarcinoma tumor. This left-sided 2x2cm lesion caused facial droop, sensory deficits, and hearing loss.

**Figure 5:**
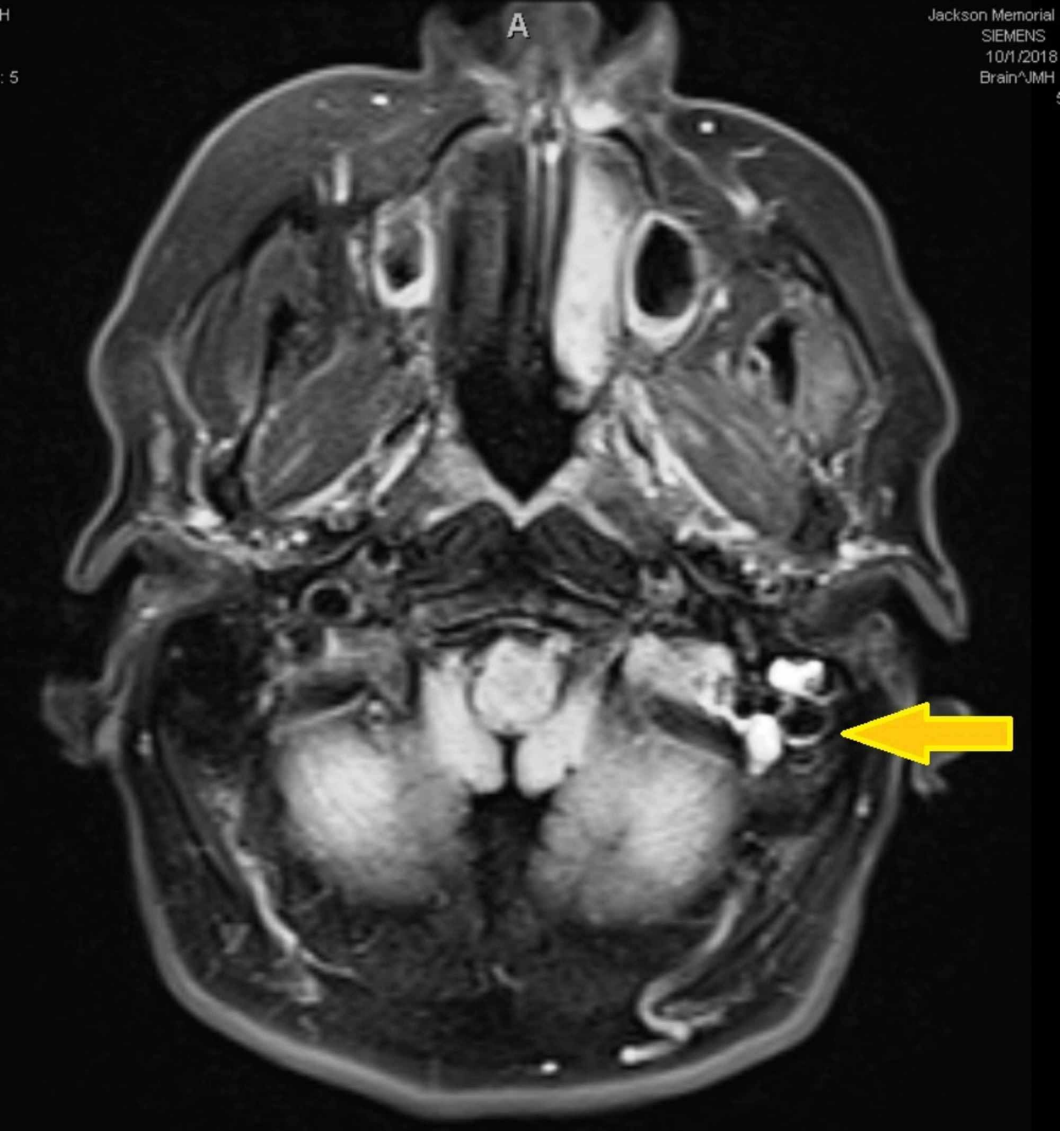
Tumor seeding along CNIII and CNV in cranial fossa and parotids. NSCLC with a neuroendocrine profile characterized by differentiation or morphology features is presumed to bear a poor prognosis [10].

In 2017, the patient came in with left CN VII neuropathy, shooting pains, sinus headaches and concerns about eyelid motor function. However, MRI of the brain and PET CT came back negative for cancer. The patient underwent multiple sinus drainage and eyelid closure surgeries. The University of Miami Neurology ordered a ‘Neocomplete Paraneoplastic Evaluation with recombx’ MRI of the base of the skull. Subsequent PET CT showed metabolically active tumoral activity in the middle cranial fossa, parotid glands, and osseous disease with a spinal nerve involvement at T6 [10]. Left-sided craniotomy revealed a metastatic poorly differentiated adenocarcinoma that extended through foramen ovale/rotundum to the infratemporal fossa and caused a left-sided facial paralysis, hearing loss, and numbness in CN V2-V3 distribution. See Figure 4 and Figure 5 for the location, morphology, and size of the tumor.

 

**Figure 6:**
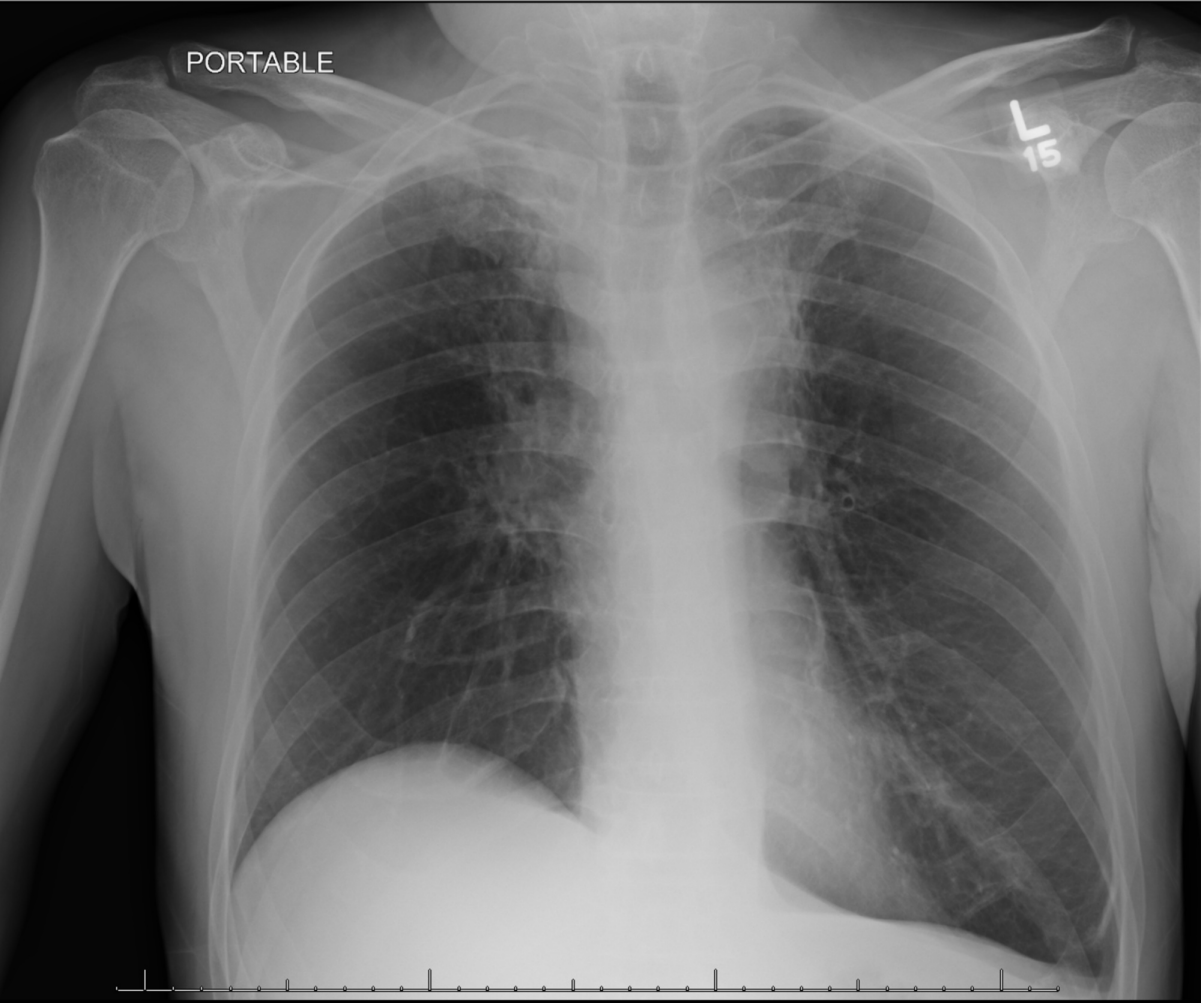
Post-radiation treatment chest x-ray showing fibrotic changes in the lung parenchyma. Chest radiography features suprahilar opacities more on the left, silhouetting the aortic arch and apical pleural thickening right more than left, that represent fibrotic apical changes due to surgery and radiation therapy. There is flattening of the left hemidiaphragm and elevation of the right. There is also pericardiac opacity in the right upper zone of the right lung.

On physical exam as of October 2018, the patient appeared well-nourished, able to close his left eye, cognitively intact but with an impaired motor aspect of speech and facial expression. Traces of dried blood in the ear, a well-healed 10-cm scar on left face and neck, and prominent bilateral tortuous chest thoracic veins were observed. The patient reported left-sided facial numbness and diminished hearing but denied any pain. Chest X-ray revealed the collapse of the lower lobe of the right lung and compensatory emphysema on the left side, but no new lung masses (Figure 6).

The patient remains asymptomatic as of his last follow-up visit in May 2019. 

## Discussion

Pathophysiology of SVC syndrome due to malignancy is related to extrinsic compression by primary tumor mass, mediastinal lymphadenopathy, or direct tumor invasion into the vasculature [3]. Collateral channels are formed to restore venous return when stenosis exceeds 60%. The site of obstruction (pre-azygos, azygos, post azygos) dictates the vascular involvement. The most efficient collateral system is the right superior intercostal and azygos circulation. For this reason, most of the patients with pre-azygos obstruction of SVC remain asymptomatic for a long period of time [4].

This patient’s initial presentation was also at an advanced stage. He responded well to standard protocols but has experienced several recurrences of disease and is not a candidate for targeted molecular therapies based on ALK/KRAS/EGFR/BRAF testing. Therefore, additional genetic profiling (CTAL4/PDL1/MSI/MMR) and immunotherapy trials are suggested as the next step at this time [7, 11]. Immunologic biomarkers (constitutional or somatic alterations in tumor cells) that might be correlated with systemic or local alterations of the immunity status observed in some patients with advanced cancer, e.g. lymphopenia, over-representation of Treg cells and dendritic cells alterations should be identified and documented to augment ongoing clinical trials [11, 12-16].

The majority of current literature on this subject describes poor clinical outcomes of NSCLC with concomitant SVCS. The natural history of this patient’s disease is remarkable in terms of metastatic sites, paraneoplastic manifestations, and a substantially prolonged lifespan. The question of whether there is anything in this patient’s metabolic or genetic blueprint that gave him this survival advantage and anything that we can synthesize remains unanswered and calls for more studies of similar cases as it will surely advance our understanding of the tumor genetics and immunotherapy, thus allowing greater benefit to future patients [6, 17].

## Conclusions

Clinicians must maintain a high index of suspicion for an uncommon presentation of a common disease. In this case, the adenocarcinoma (an epithelial cell tumor) of NSCLC followed the manifestation, spread, and mutation pattern of a small cell lung carcinoma (a neuroendocrine cell tumor). Cancer can be treated as a chronic disease rather than a terminal illness. Thanks to advances in immunology and histochemistry, onco-pharmacology is rapidly evolving and is becoming more effective in treating advanced cancer and previously uncontrollable tumors. Clinicians should encourage patients to contribute to the International Cancer Genome Consortium studies that aim to develop a map of immunologic and genetic profiles for all types of advanced malignant tumors and biomarkers. Several state agencies in the United States collect some of the abovementioned evidence. However, a simple standardized platform for donating patient data locally to maximize the outreach and to subsequently merge this information globally is yet to be developed. Nonetheless, every new scrutinized patient case brings us one step closer to another inspiring therapeutic breakthrough.
